# Narrowband UVB treatment is highly effective and causes a strong reduction in the use of steroid and other creams in psoriasis patients in clinical practice

**DOI:** 10.1371/journal.pone.0181813

**Published:** 2017-08-03

**Authors:** John Foerster, Kirsty Boswell, Jonathan West, Heather Cameron, Colin Fleming, Sally Ibbotson, Robert Dawe

**Affiliations:** 1 University of Dundee, Medical School, Dundee, Scotland; 2 Department of Dermatology and Photobiology, NHS Tayside, Dundee, Scotland; Kinki Daigaku, JAPAN

## Abstract

**Background:**

Narrowband NB-UVB phototherapy (NB-UVB) is an effective treatment for psoriasis, as demonstrated by clinical trials. However, due to required infrastructure and need for treatment attendance opinions on the value of offering this treatment in routine practice vary. AIMS: To provide high quality large-scale and long-term data on the efficacy of NB-UVB for psoriasis under real-world conditions in order to assist in management decisions.

**Methods:**

The following resources were employed: (1) complete and prospectively recorded prescription drug records for a population of 420,000 marked by low demographic mobility, (2) prospectively recorded clinical treatment outcomes for all NB-UVB treatment episodes occurring in the local population; (3) complete dermatology electronic treatment records of all psoriasis patients, allowing cross-validation of diagnoses and treatment records. Using these data sets, we analysed all first-ever initial NB-UVB treatment episodes occurring over 79 months (n = 1749) for both clinical outcomes and the effect of NB-UVB on the use of topical treatments for psoriasis.

**Results:**

Around 75% of patients both achieved a status of “clear/minimal disease” and used fewer topical treatments. NB-UVB treatment led to a strong reduction for both steroid creams (25%) and psoriasis-specific topicals, e.g. vitamin-D products (30%) during the 12-month period following NB-UVB treatment. The effects measured were specific as no effect of NB-UVB was noted on drug prescriptions unrelated to psoriasis. Results were independent of individuals administering and/or scoring treatment, as they were highly similar between four geographically separate locations.

**Conclusions:**

NB-UVB treatment is highly effective and leads to a remarkable reduction in the need for topical cream treatments for a period of at least 12 months.

## Introduction

Narrowband ultraviolet B phototherapy (NB-UVB) has been shown to be effective in psoriasis in clinical trials [1,2] and is a cornerstone of treatment (reviewed in [3,4]). Nevertheless, access to NB-UVB is not universally provided since both the required infrastructure, as well as the need to attend for treatment are viewed as severe limitations. Therefore, robust data about its efficacy under real-world conditions would be valuable to inform decisions as to whether efforts to increase access to NB-UVB, are appropriate in times of limited resources.

The assessment of efficacy out with controlled interventional studies is fraught with a number of well-known issues. These include first and foremost recording bias of data as well as selection bias in terms of the observational study cohort. To try and address these limitations, we have recently described a locally available set of factors allowing almost complete IT-based capture of medical interventions, and applied these toward defining efficacy and safety of methotrexate treatment under real world conditions [5]. These factors include comprehensive electronic capture of all prescription drugs and a stable population marked by low demographic mobility and minimal private treatment out with the NHS. This provides a nearly complete set of treatment records for a population of 420,000. In the case of phototherapy, an additional factor is that clinical outcomes for all treatments were recorded prospectively. We here apply these resources to define outcomes of NB-UVB in psoriasis.

Randomized prospective trials report outcomes for psoriasis treatments, predominantly PASI and PGA scores, in a blinded manner to establish efficacy. By contrast, in routine clinical care, the aim of outcome monitoring is not to prove efficacy as such, but to establish efficacy *and* the degree of benefit *for a given patient* which helps to guide the appropriateness of repeat treatment in future flares. Therefore, outcomes are not recorded in a blinded manner, and also take into consideration patients’ subjective assessment (see below for further details), yielding a combined assessor- and patient-reported outcome. Therefore, it is of fundamental interest to determine whether the semi quantitative outcome scale employed here can be validated by independent objective outcomes.

Using our access to drug prescribing data, we here report the impact of NB-UVB treatment on the use of psoriasis prescription drugs over time. The results validate the operator-recorded clinical outcome scores. Moreover, the data show that NB-UVB treatment achieves a remarkable reduction in the use of steroid creams, as well as other topical treatments, representing a significant secondary benefit both to patients as well as health care providers. Notably, using data from four geographically independent treatment sites we show that outcomes are independent of local factors such as patient demographic, or staff experience.

## Methods

### Ethics statement

All data generated in this study were obtained in accordance with the Declaration of Helsinki and in compliance with local governance approval regulations (Approval by: NHS Tayside Caldicott guardian committee, approval nr CSAppJF2101).

### STROBE statement

This is an observational cohort study. In accordance with the STROBE checklist [6], background and objectives are specified in the Introduction, design, setting, participants, variables, data sources, quantitative variables, statistical methods, used, and bias (reporting as well as selection) are discussed below and in Results. All other elements listed in the STROBE checklist, specifically Limitations, are listed in Results and Discussion.

### Patient cohort

Initial screening of patients was performed through identification of all patients who were listed as having a diagnosis of ‘*psoriasis*’ in the systematic diagnostic records contained within Photosys, the database used by Photonet (www.photonet.scot.nhs.uk). Photonet is the national managed clinical network for phototherapy in Scotland, and Photosys contains records of all courses of NHS phototherapy administered throughout Scotland. The observational interval was defined as between 01/01/08 and 01/04/15 (79 months). This observational window was chosen as the maximum interval available where all pertinent data (NB-UVB treatment outcomes, prescribing data, demography-linked databases) were accessible for all individual treatment locations in all of the four treatment centres coordinated by the Tayside Department of Dermatology (Dundee, St Andrews, Perth and Stracathro). In accordance with local governance regulation, patient identifiable information was anonymized by the Health Informatics Centre Tayside (HIC; www.farrinstitute.org).

### Definition and limitation of treatment episodes

The resultant crude cohort contained all patients having undergone NB-UVB for a diagnosis of psoriasis within the specified observational period. This treatment dataset was further limited to involve only first-ever treatment episodes for two reasons. Firstly, this limitation excluded potential effects on drug prescribing that could theoretically have been due to long-term remissions effected by prior treatment episodes. Secondly, it minimized a potential bias on the frequency of non- responders. Thus, the subgroups of early relapse patients (defined as having undergone additional NB-UVB treatment in less than 1 year after starting the initial treatment) would have been expected to be artificially increased if 2^nd^ or 3rd NB-UVB treatment episodes had been included. NB-UVB treatment was considered to have occurred if ≥ 10 individual treatment sessions had been administered (see S2 Table).

### Cohort validation and refinement

The raw cohort detailed above comprised of 1802 patients. For the purpose of the present study, patients with a concurrent diagnosis of photodermatosis (e.g. polymorphic light eruption) were omitted (n = 53 equivalent to 0.99%) in order to exclude the possibility that NB-UVB had, in fact, not been administered to treat psoriasis. Further cross-validation with local treatment records identified n = 9 (0.5%) patients, who had a correctly assigned diagnosis of psoriasis but no documentation of NB-UVB therapy performed within the observational window, and were excluded accordingly. Parallel cross-check with the HIC-provided prescribing database identified n = 18 (2.0%) patients were identified as having no prescribing records available (reasons unclear) and were therefore removed from the study, resulting in a finalised cohort size of n = 1749 patients. A final quality control check was applied by screening the resultant database of patients against the local dermatology database Dermabase, which harbours a structured diagnostic index for all patients treated within the department. This cross-check did not identify any patients mis-assigned with a diagnosis of psoriasis in Photosys. In addition, the Dermabase check revealed that n = 4 patients (0.2%) in the final cohort had a concurrent diagnosis of atopic dermatitis). Although this would represent an important confounder with respect to steroid treatment, omission of these patients did not change any of the outcomes present in the Results section, presumably due to the small number of patients.

### Prescribing information, clinical profiling and data refinement

The cohort described above was linked to the electronic drug prescribing dataset as previously described [5]. Initial application of this process returned 422,175 prescription incidents (average of 38 prescriptions per patient per year). 266,954 were prescribed unrelated to psoriasis. Among these, prescriptions for the most common, unrelated co-morbidities identified- hypertension and depression- were recorded separately to serve as a control set for prescriptions made out unrelated to psoriasis and to define co-morbidity status (see below). In addition, antihistamine prescriptions were also sub-analysed, in order to test a possible dependence on NB-UVB treatment. All remaining psoriasis-unrelated prescriptions were designated as “other” and also quantified as a further independent set of internal control prescriptions. The remainder of prescriptions were classified and quantified as detailed below.

### Quantification of psoriasis treatments

Psoriasis-relevant prescriptions made out to patients in the cohort were inventoried as described by their British National Formulary (BNF) code as follows: emollients (13.2.1), steroid creams (13.4), Acitretin (13.5.2), Methotrexate (8.1.3 + 10.1.3), Cyclosporine (8.2.2) and topical treatments (13.5.2). Prescriptions were then filtered for occurrence within either the pre- or post-treatment observational windows for each individual patient. The category of “psoriasis-specific” topical treatments included the BNF code 13.5.2 and comprises calcipotriol, calcipotriol with betamethasone, calcitriol, coal tar products, dithranol, salicylic acid compounds, tacalcitol, as well as tazarotene.

### Assignment of comorbidity status based on medical treatment

Concurrent diagnoses of hypertension (HTN) and depression were inferred from analysis of prescribing records based on the specific BNF codes relating to medications indicated in the treatment of these conditions, for antidepressants (BNF 4.3) and for anti-hypertensives (BNF codes 2.2.1, 2.5.1, 2.5.2, 2.5.3, 2.5.4 or 2.5.5), respectively, as described previously [5]. In order to exclude erroneous assignment of a diagnosis based on short-term or spurious prescription, a concurrent diagnosis of depression was only assigned to patient who had received a minimum of 2 separate prescription for either diagnosis during the 12 month pre-treatment interval. Diabetes status was assigned based on linkage to the SCI-DIABETES database held by HIC, as previously described [5].

### Study design

Ideally, a prospective study design would have been chosen to compare treatment outcomes between randomized cohorts assigned to non-NB-UVB or NB-UVB treatment. As this study design was not feasible we devised a design such that ‘before’ vs. ‘after’ NB-UVB psoriasis treatments administered to patients would be quantified (see Results). The study contains both retrospective and prospective design elements. Thus, the clinical treatment outcomes within the Photosys database are continuously recorded prospectively. Likewise, the prescribing of drugs is electronically captured prospectively at the time of prescribing. By contrast, the extraction and analysis of data as presented here has been performed retrospectively.

Theoretically, any change of treatments dispensed ‘after’ NB-UVB could be purely by chance, reflecting the natural fluctuations in the course of an individual patient’s disease course rather than the effect of NB-UVB. To address this, the study design included multiple sets of built-in internal controls datasets by capturing both psoriasis-related as well as–unrelated drug prescribing data. Finally, in order to further reduce confounding effects, we selected only the initial NB-UVB treatment episode for any given patient (see Results). The disadvantage of this choice was the reduction of sample size and, importantly, an inability to describe intra-individual variability of response to treatment across multiple treatment courses. The advantage, however, was the elimination of selection bias such that patients undergoing second or multiple treatment episodes would be assumed less likely to have achieved a good response at first treatment. In addition, this limitation minimized any potential effects on drug prescribing related to prior NB-UVB treatments on the current treatment episode (see S1 Table).

### Statistical methods and treatment of incomplete data

The choice of statistical test applied to ascertain statistical significance is indicated in the legend of the respective figure summarizing data, as well as individual tables. Data completeness (treatment episodes, prescription data, clinical treatment outcomes) was accounted for by selection of observational window and exclusion of treatment episodes, as detailed above (cohort refinement).

## Results

### The NB-UVB treatment cohort

As detailed in Methods, we collected data from all consecutive patients receiving NB-UVB-treatment within an observational period that was limited by the availability of complete datasets. The resultant clinical characteristics are summarized in Table 1. As expected, the cohort as such mirrors the main co-morbidities observed for psoriasis patients in the BADBIR cohort, hypertension, diabetes, depression, therefore suggesting that efficacy data obtained in this cohort may be informative for the UK, as well as other populations of similar genetic make-up. In line with local approaches to psoriasis treatment pathways, the cohort represents an almost systemic-treatment naïve sample with only 4% having received methotrexate prior to NB-UVB. This makes it unlikely that any observed treatment outcomes are confounded by prior or concurrent on-going systemic treatments. The cohort therefore represents the total of consecutive NB-UVB treatment episodes initiated for psoriasis patients deemed inadequately controlled by topical treatment in an observational window of 79 months. On a population basis, this translates into an incidence of 5.2 treatment episodes per month per 100,000.

**Table 1:** NB-UVB treatment cohort^1^.

Clinical Characteristics	Number of Patients	Percentage of Patients (%)
	Total (n = 1749)	%
Female	869	49.7
Median age (range) at NB-UVB1	48 (5–88)	
Diabetes	160	9.2
HTN	334	19.1
Depression	301	17.2
Pre-treatment Methotrexate	50	2.9
Pre-treatment Acitretin	4	0.2
Pre-treatment Cyclosporine	1	0.1

^1^Co-morbidities were assigned based on indication-specific drug prescribing (hypertension and depression), or based on disease-specific database linkage (diabetes), respectively, as detailed in Methods.

### Selection of pre- and post-treatment observational windows

**Fig 1:**
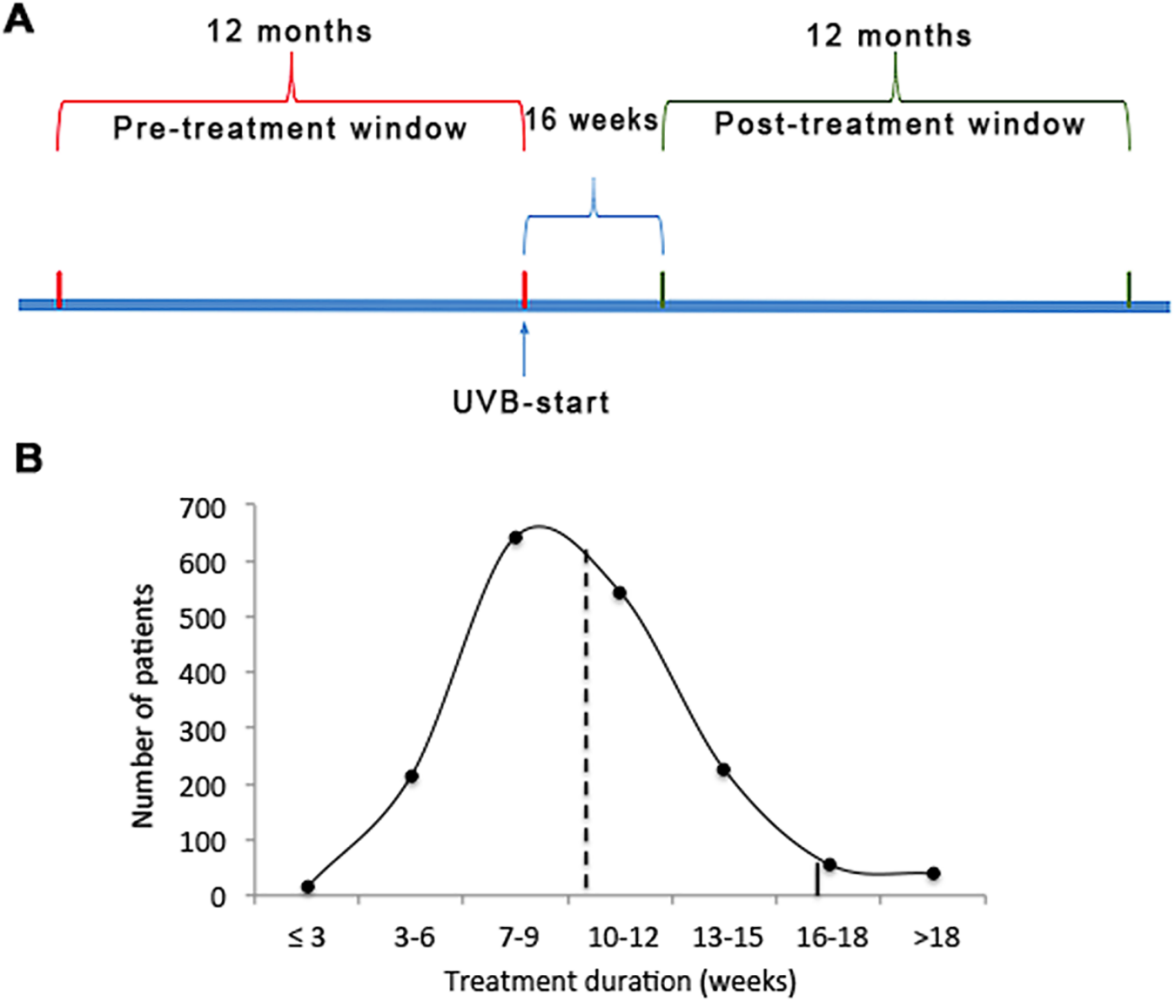
Study design. A. Timeline illustrating the 12-month pre-treatment and post-treatment periods, respectively, as well as the 16-week treatment interval (for details, see text). B. The distribution of the duration of NB-UVB treatment episodes across all patients. Dashed line: median (9.3 weeks). Solid line indicates the 16-week cut-off selected for beginning of the “post-treatment” observational window with n = 74 (4.2% of all) treatments exceeding this cut-off.

A key methodological aspect in the quantification of topical treatments applied before-versus-after NB-UVB therapy in psoriasis is the duration of the observational window. The shorter the window, the larger the effect of natural fluctuations in disease activity. The longer the post-treatment window, the greater the risk that subsequent UV- (or other systemic treatments) will confound the data, thereby also indirectly limiting the pre-treatment window. Finally, the choice of starting point for the post-treatment observational window is critical as, under conditions of routine clinical practice, treatment duration will vary considerably between patients, depending on their response. Taking into account these factors, we selected a twelve-months pre- versus post-treatment window (Fig 1A) in order to satisfy two key protocol requirements. First, a 12 months window is sufficiently long to minimize seasonal fluctuations in psoriasis prescriptions filled. Second, analysis of the data showed that this observational window was, conversely, short enough to minimize the occurrence of additional subsequent NB-UVB treatment courses that would represent potential confounders (rate of 11.1% of 2^nd^ UV treatment commenced at < 1 year after 1^st^ UV treatment). To further safeguard against this confounder, outcomes from the study cohort were analysed both with and without inclusion of this sub-cohort of patients. The impact of the 2nd UV-treatment episode initiated within the post treatment interval could therefore be gauged (see below and S1 Table).

The time point chosen for the beginning of the post-treatment window was chosen based on the distribution of NB-UVB treatment sessions administered for all treatment episodes across the entire cohort (Fig 1B). Calculation of drug prescriptions based on the actual time point of completion in each individual treatment course was not possible due to limitations in data linkage across absolute points in time. Therefore, we selected “16 weeks post treatment initiation” as beginning of the post-treatment observational window based on the observation that only very few (4.2%) of all treatments exceeded this treatment duration (Fig 1B, solid line), while at the same time minimizing the number of weeks scored as “post treatment” in patients that completed their treatments in a shorter period of time. Using this cut-off, the post-treatment window in fact begins at an average of 6.4 ± 3.5 weeks after completion of the NB-UVB course across all patients (S2 Table). Therefore, selection of this post-treatment window represents a conservative approach since a benefit of NB-UVB treatment on drug prescribing would be expected to be most pronounced immediately after completion of treatment.

### Overall treatment outcomes

**Fig 2:**
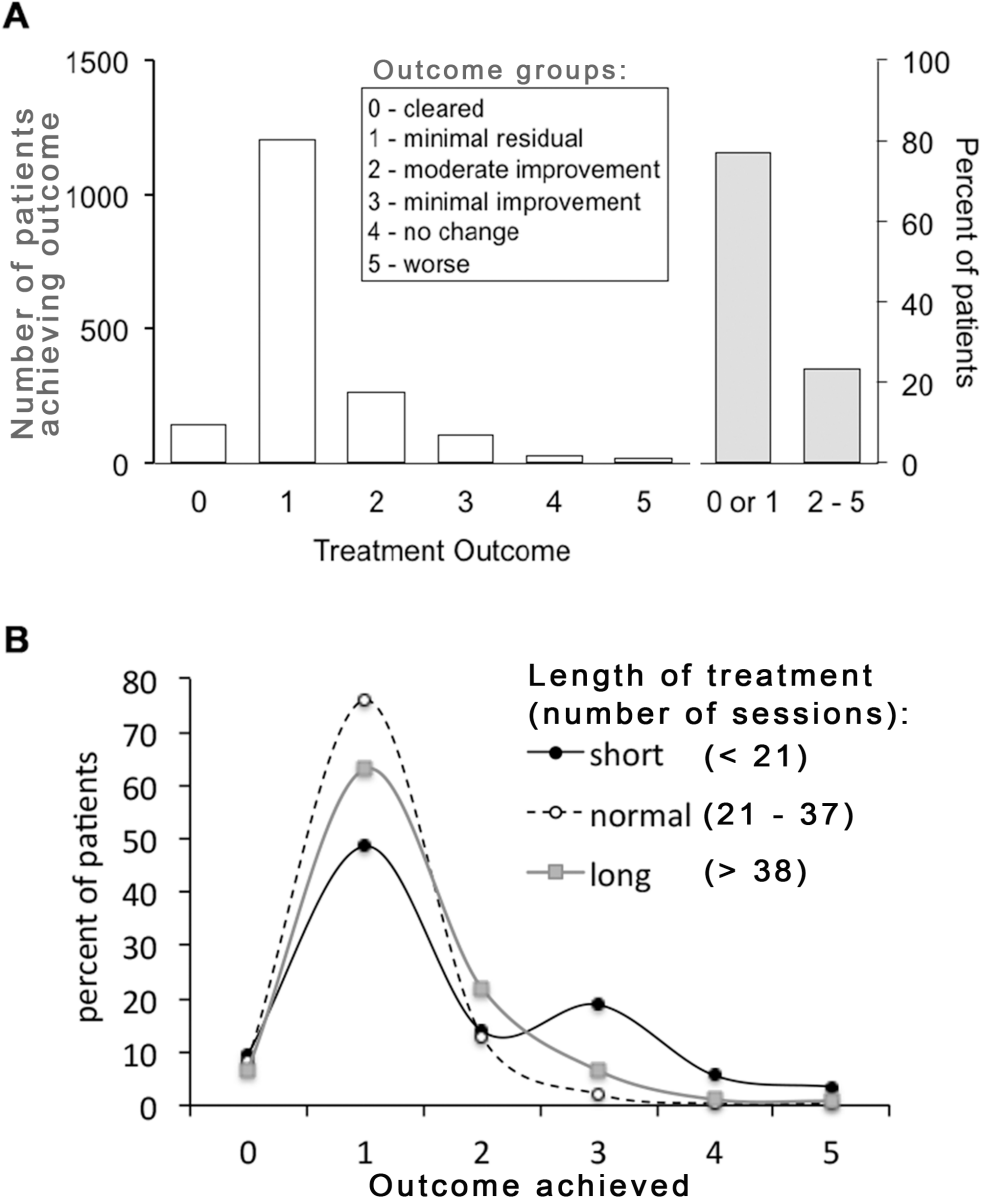
The clinical outcome of NB-UVB-phototherapy on psoriasis. A. Semi-quantitative outcome classes recorded for all phototherapy treatments (n = 1749), shown in number per patients (left y-axis), and as percentage of patients achieving either outcomes 0/1 or 2–5, respectively (right y-axis). B Outcomes achieved as a function of treatment duration, showing the percentage of patients in the lower 20^th^ percentile (“short” = less than 21 sessions), in the 21-80^th^ percentile (“normal’, 21–37 sessions), and above the 80^th^ percentile (“long”), respectively. P < 0.0001 for the difference across all three groups (chi-square).

Fig 2 shows the global recorded treatment outcomes across the entire cohort. Outcomes were recorded on a semi-quantitative scale ranging from 0 (clear) to 5 (worse), as shown in the figure. The scale represents a hybrid between a static assessment for outcomes 1 and, largely overlapping with PGA scales commonly used in controlled trials, and an assessment relative to baseline for outcomes 2–5, respectively. The hybrid nature of the scale is owed to its function not to prove efficacy (in contrast to a randomized study) but to guide clinical management in routine clinical care. To optimize its utility for this purpose, the scale is also not strictly either patient-reported or assessor-reported but selected as consensus between patient and operator. Thus, assignments of scores 0/1 essentially imply that the patient would be willing to contemplate future NB-UVB treatment episodes. By contrast, scores 3–5 essentially state that UV was ineffective, making a subsequent repeat trial less likely. Outcomes 0/1 (“clear”, “minimal residual disease”) largely overlap with a score of PGA 0/1. This is the outcome recorded (unblinded) for approx. 75% of patients (Fig 2A, right).

### Effect of treatment duration on clinical outcome

We next analysed the clinical outcomes for three sub-cohorts: the 20% of patients receiving fewest and most treatment sessions, respectively, and the remaining 60% of patients falling in between. As shown in Fig 2B, both the “short”, as well as the “long” sub-cohorts of patients achieved significantly worse outcomes. For the “short” sub-cohort, in particular, there is a sizeable number of patients (18% of this sub-cohort) achieving only “minimal improvement”. In all likelihood, this represents a subgroup of patients where treatment was prematurely discontinued for any number of non-recorded reasons. Accordingly, the number of patients achieving only this outcome is even greater (28%) in the lowest 10^th^ percentile (patients treated for <18 sessions). Likewise, in the “long” sub-cohort, 9.4% of patients only achieve outcomes 3–5 vs. 2.8% in the “normal” subgroup. These represent patients where treatment is continued in the hope of delayed-onset clearance without the desired outcome. Importantly, these data demonstrate that the complete cohort includes both prematurely discontinued as well as non-responding patient groups. We conclude that the prospective recording of clinical outcomes therefore yields data free of bias toward responding patients, rather analogous to intention-to-treat analysis data in prospective randomized trials.

### Effect of NB-UVB treatment on prescription of psoriasis-related drugs

**Fig 3:**
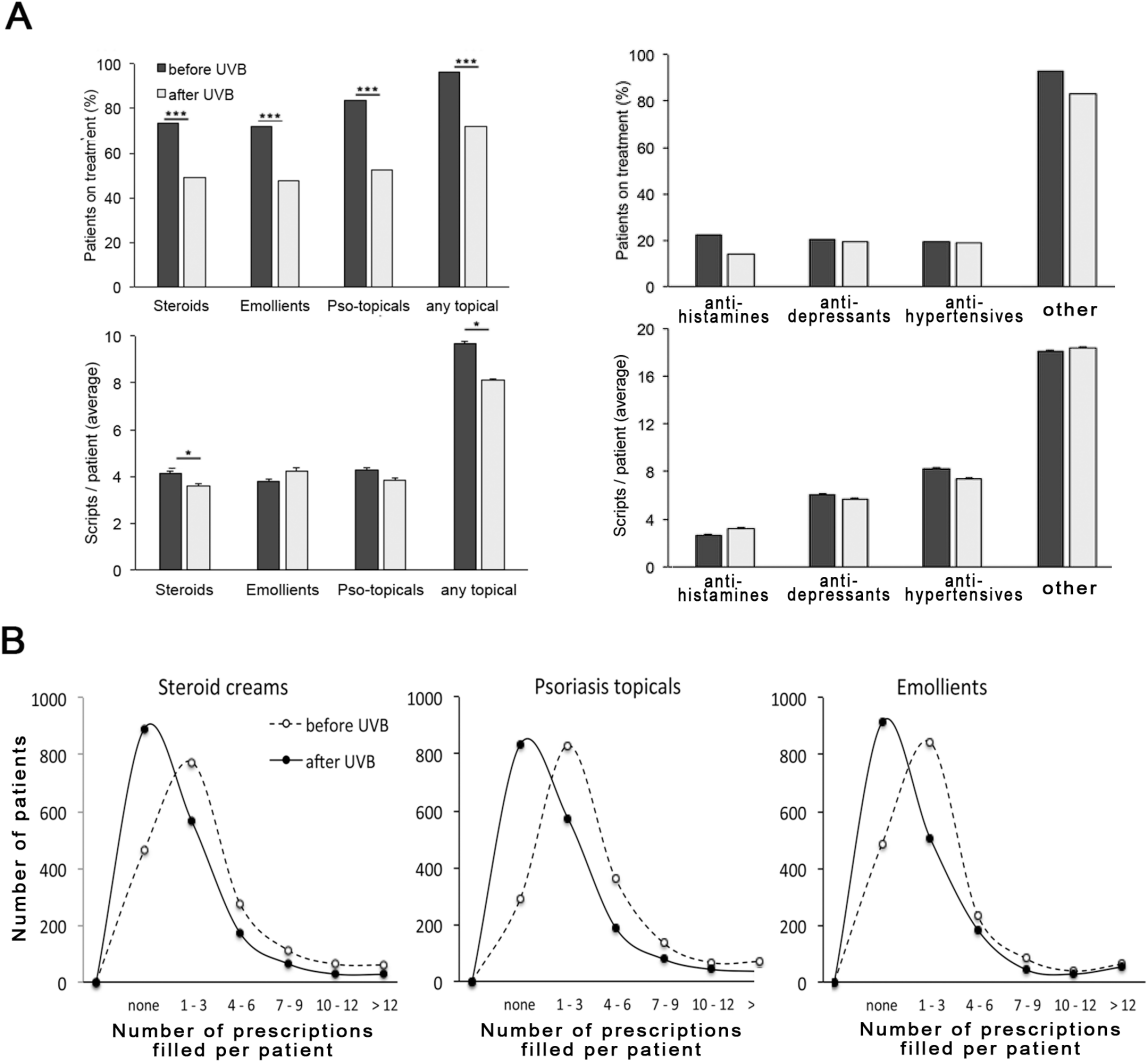
The effect of NB-UVB on drug prescribing. A. Top: Prescription of psoriasis-related (left) and–unrelated (right) drugs in psoriasis patients before (dark) and after (light shaded) NB-UVB phototherapy. H1 –antihistamine, Depr–anti-depressive drugs, HTN–antihypertensive drugs (for details see Methods). Shown is the percentage of patients on treatment. *** p <0.0001 (Qui-square); bottom: the number of scripts per patient in patients receiving treatment (data shown represent average ± s.e.m.). * p < 0.01 in a two-sided T-test. B. Histogram plots showing the overall distribution of prescriptions filled for the topical treatment classes psoriasis before (solid) and after NB-UVB (dashed), respectively.

We next assessed the outcome of NB-UVB treatment independently of clinically recorded outcomes by quantifying the prescription of drugs before and after the treatment course. As shown in Fig 3 (top), we observed a highly significant reduction in the number of patients receiving psoriasis-related topical prescriptions within a year after completion of NB-UVB treatment, while no such effect was noted for blood pressure, depression, or other drug prescriptions unrelated to psoriasis (Fig 3, top right). The effects were remarkable, with 24% fewer patients requiring steroid creams and 31% fewer patients psoriasis-specific topicals (Table 2). Interestingly, there was a moderate, but statistically significant reduction of patients receiving anti-histamine drugs (22.4% before vs. 14.0% after NB-UVB). This may reflect the subset of patients where psoriasis causes pruritus and/or possibly a concomitant effect of NB-UVB treatment on co-existent but psoriasis-independent pruritus. In addition, we noted a significant reduction in the number of scripts filled per each patient undergoing any given treatment for steroid creams (4.1 ± 0.1 vs. 3.6 ± 0.1, average ± s.e.m.), as well as psoriasis-specific topicals (4.3 ± 0.1 vs. 3.8 ± 0.1) whereas no such effect was noted for any other drug class. Overall, 71% of patients exhibited a decrease in prescriptions compared to 29% showing an increase (S1 Fig), which is comparable to the ratio of patients achieving outcome 0/1 vs. 3–5 (76.8 vs. 23.2%, see above, Fig 1A).

**Table 2:** The change in the number of patients receiving topical psoriasis- treatments after one course of NB-UVB phototherapy^1^.

	Before NB-UVB		After NB-UVB		Percent Change
	N	%	N	%	
Steroid creams	1287	73.6	859	49.1	-24.5
Psoriasis topicals	1461	83.5	917	52.4	-31.1
Emollients	1262	72.2	835	47.4	-24.4
Any topical	1681	96.1	1260	72.0	-24.1

^1^ N = 1749.

### The effect of clinical treatment outcome on the impact of NB-UVB on drug prescription

**Fig 4:**
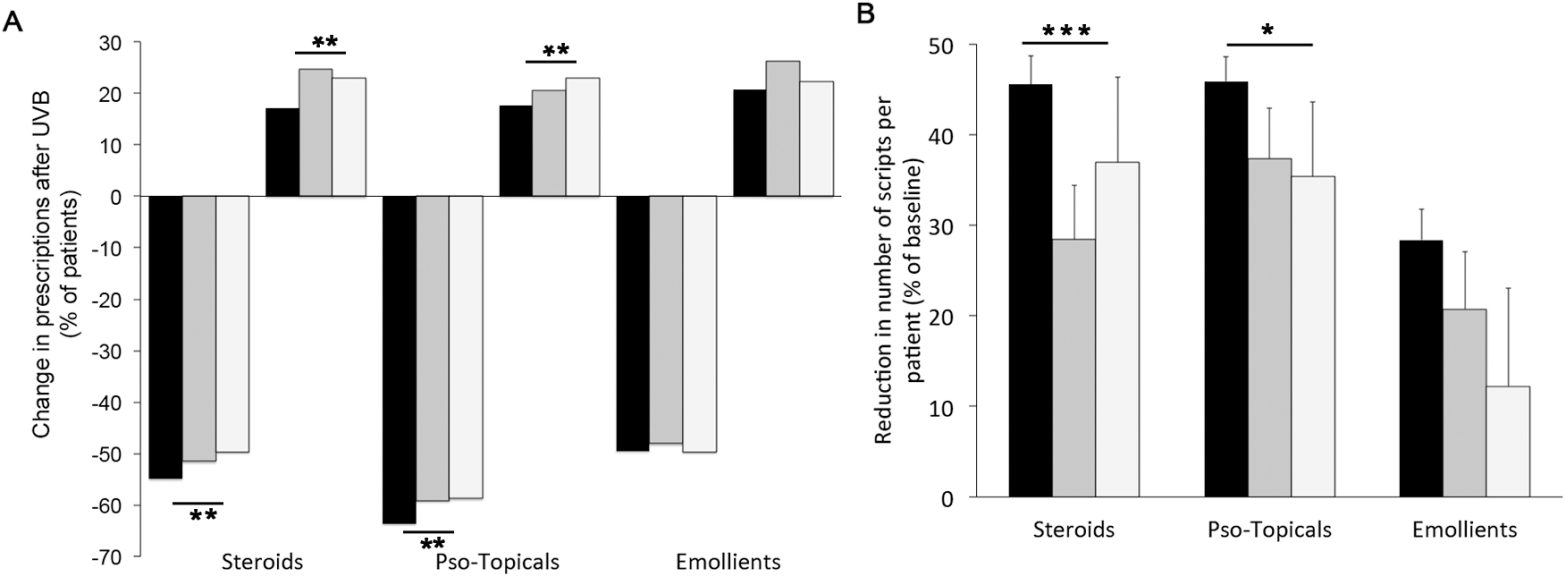
The change in psoriasis-specific drug prescribing before vs. after NB-UVB phototherapy in patients achieving different clinical outcomes. A. The percentage of patients in each outcome class showing either a decrease, or an increase in the number of prescriptions for each drug class indicated. Black columns: outcomes 0/1 (“clear”, “minimal residual disease”), dark grey: outcome 2 (“moderate clearance”), light grey: outcomes 3/4/5 (“minimal change”, “no change”, “worsening”). B The change in prescriptions made per patient, compared between after vs. before NB-UVB treatment (detailed in Table 3), expressed as percentage reduction compared to baseline, for each of the outcome classes, as in A. *** p < 0.0001, ** p < 0.001, * p < 0.01 (chi-square for the difference between the three outcome classes).

There was no direct correlation between recorded treatment outcomes and change in prescriptions after NB-UVB (not shown). We therefore asked if the magnitude of changes in psoriasis-specific drug prescription after NB-UVB treatment differs between subgroups of patients achieving different outcomes. To this end, we combined the outcome groups into three classes: clear/almost clear (outcome groups 0/1), “moderate” (outcome group 2), and “minimal change/ no change/ worse” (outcome groups 3/4/5), respectively. This comparison is somewhat limited since the “moderate” group likely is a rather heterogeneous group. We did not detect statistically significant differences in the decrease of patient numbers requiring topical treatment between the outcome classes (S1 Table). However, the reduction of prescriptions filled for each patient was more pronounced in magnitude (Fig 4A), as well as more statistically significant (Table 3) in patients achieving clinical outcomes 0/1.

**Table 3:** The change in prescriptions per patient after NB-UVB phototherapy^1^.

	Steroids		Pso-Topicals		Emollients	
Outcome group	Before	after	before	after	before	after
0/1	3.0 ± 4.3	1.6 ± 3.7**	3.5 ± 4.0	1.9 ± 3.7*	2.6 ± 4.0	1.9 ± 4.5**
2	3.5 ± 4.0	2.5 ± 4.0**	4.0 ± 4.4	2.5 ± 4.0	3.2 ± 5.2	2.6 ± 5.2*
3–5	2.9 ± 3.5	1.8 ± 3.2*	3.0 ± 3.2	1.9 ± 3.5	2.7 ± 3.7	2.4 ± 5.7

^1^ Outcome groups are “clear/minimal residual disease” (0/1), “moderate clearance” (2), “minimal improvement, no change, worsening” (3,4,5), respectively. Data shown represent average ± s.d. for n– 1343 (outcome groups 0/1), n = 262 (outcome group 2), and n = 143 (outcome groups 3/4/5), respectively.

* p < 0.05 (two-tailed paired t-test)

** p < 0.001, bold-set: p < 10^−10^

Moreover, the overall reduction in prescriptions filled for each of the psoriasis treatment classes shown above (Fig 3) in fact represents an aggregate of patients exhibiting a sliding scale of decreased, unchanged, or even increased drug prescriptions (detailed in the histogram in S1 Fig). We therefore compared the number of patients in each of the three clinical outcome classes showing either a decrease or increase in prescriptions filled for each of the treatment classes. Again, we observed consistently that outcome class 0/1 exhibited the greatest percentage of patients showing a decreased number of prescription, as well as the lowest percentage of patients requiring more prescriptions after NB-UVB treatment for each treatment class (Fig 4B). These data strongly suggest that the observed reductions in psoriasis-specific drug prescriptions are most prominent in those patients that had been recorded as achieving greatest treatment benefit at the end of their NB-UVB course.

The quantification of drug prescription, as detailed above, is a substantively different outcome from clinical skin assessment since the former represents the integral of changes occurring over a 12-months period, while the latter is a momentary score. Therefore, drug prescribing in effect measures a combination between the degree of disease remission achieved at the conclusion of UV-treatment as well as duration of remission. Bearing these differences in mind, the results detailed above that the highly statistically significant reduction of drug prescriptions observed only for psoriasis drugs after NB-UVB treatment is most prominent in patients recorded as exhibiting the best clinical outcome. As such, these data suggest that the non-blinded, non-placebo controlled semi-quantitative skin assessment applied at the conclusion of phototherapy under routine clinical conditions is validated by objective, assessor-independent quantification of psoriasis-specific drug prescriptions.

### Global treatment outcomes by treatment location

**Fig 5:**
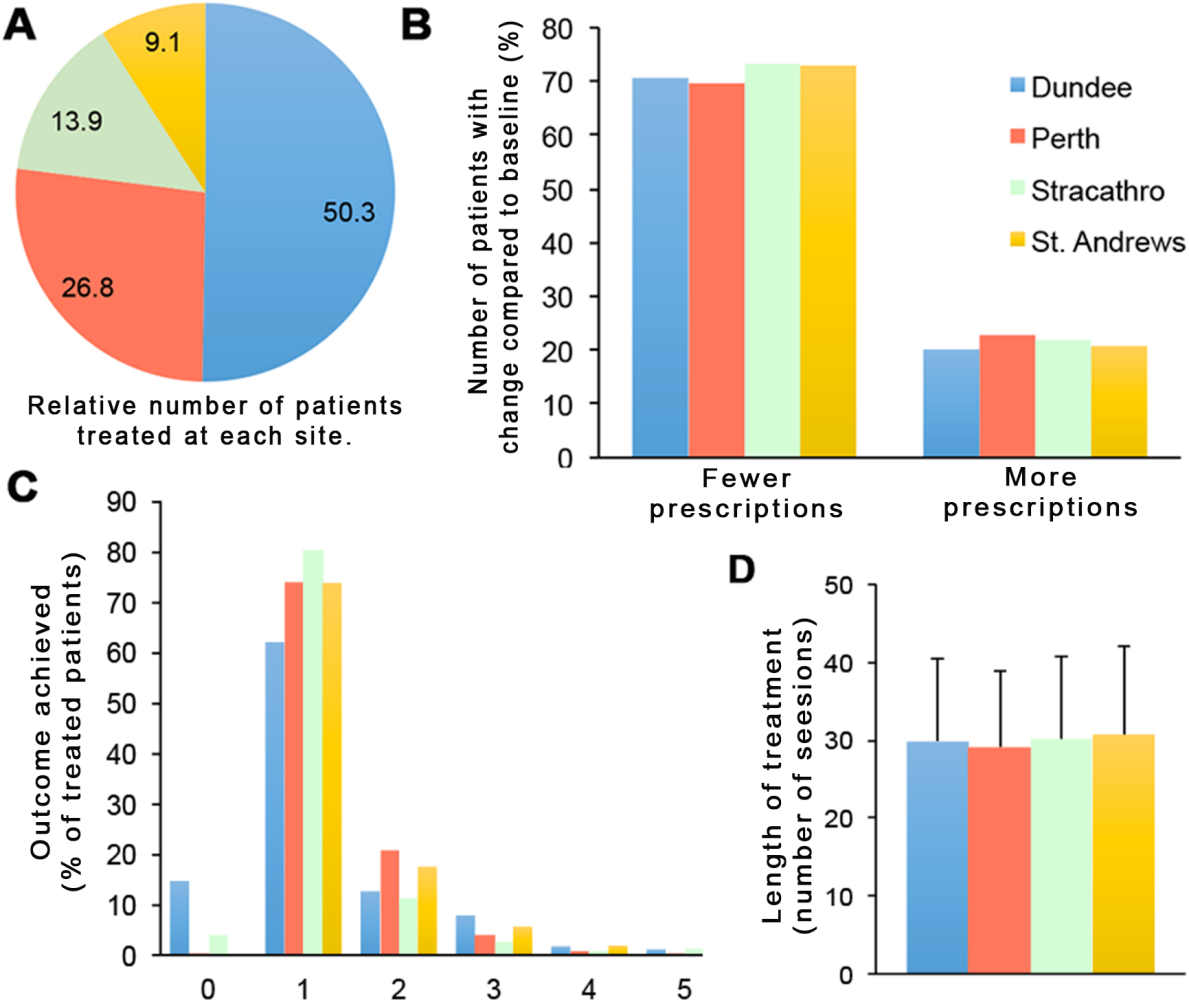
Clinical outcomes of NB-UVB treatment for psoriasis, and change in topical prescriptions made out for psoriasis, at four independent treatment sites, as indicated (b). A, pie chart showing the percentage of the overall treatment cohort (n = 1749) receiving treatment at each individual site. B, The percentage of patients at each site exhibiting either a reduction, or increase in the number of topical prescriptions made out for psoriasis after compared to before NB-UVB treatment. C, The clinical outcome recorded at each site, shown as percent of patients assessed for each outcome. (Outcome classes are identical to those shown in Fig 2, above). D, The number of treatment session administered per NB-UVB course (average ± s.d.)

One common limitation of real-world observational studies is the lack of information about the effect of local factors affecting efficacy rates. The present sample allowed us to assess the impact of this variable, since UV-treatment in the catchment of the coordinating department is in fact administered across four geographically distinct and independent locations characterized by differences in urban versus rural patient catchment, the overall throughput of treatment episodes, as well as different staff. These data are summarized in Fig 5. As evident from Fig 5B and 5C, respectively, neither the differences in topical creams prescribed for psoriasis in all individuals patients before vs. after NB-UVB nor the clinically assessed treatment outcomes varied significantly between the four independent treatment sites. Both the overall fraction of patients assessed as “clear/minimal disease”, as well as the fraction of patients achieving an overall reduction of topical cream prescriptions are strikingly similar between all treatment sites. The number of sessions administered per NB-UVB treatment course is also virtually identical (Fig 5D). These data suggest that the observed efficacy rates are not primarily limited to a large centre characterized by high treatment throughput or individually varying clinical assessment.

## Discussion

We here show that a single treatment course with narrowband NB-UVB has a significant impact on the treatment of psoriasis with topical creams with almost one-third of patients no longer requiring psoriasis-specific topicals and one-quarter of patients no longer needing steroid creams. The results are unlikely to be an artefact, since psoriasis-unrelated drug prescribing is not affected by NB-UVB. (As this is a largely systemic-drug naïve cohort, the percentage of patients receiving systemic treatment for psoriasis is low, see S3 Table.) Apart from our previous study on methotrexate [5], to our knowledge, this is the first time any psoriasis treatment has been shown to effect a quantifiable benefit on the use of topical treatments.

Our study has some important limitations. Thus, we only analysed first-ever initial treatment episodes of NB-UVB. It is possible that efficacy data may fluctuate intra-individually between subsequent treatment episodes. Second, the study design as such is observational. Although both clinical treatment outcomes as well as drug prescribing are recorded prospectively (unbiased), the analysis as such is retrospective, unblinded, and not placebo-controlled.

One of the interesting aspects of this study is the performance of a semi-quantitative outcome measure, which has been in use in Photonet for several years. The scale used (0–5) is not blinded and, importantly, is a hybrid combining a static assessment (outcomes 0,1), as well as assessments relative to baseline (outcomes 2–5). For obvious reasons, such a score would be inappropriate in a randomized controlled trial aiming to prove efficacy of a treatment. However, in clinical practice, the score combines ease of use with a guiding element informing future treatment of individual patients (‘repeat NB-UVB’ vs. ‘try something else’). Remarkably, despite its limitations, the data presented here do show that the use of this scale is largely mirrored by the effect of NB-UVB on drug prescribing, with approx. 75% of patients achieving a “clear/minimal disease”, and a reduction in prescriptions, respectively (Figs 2A and 4A). Moreover, the reduction in psoriasis prescribing is more pronounced in those patients achieving a better clinical outcome. Therefore, it would appear that in clinical practice, blinding as such is not required in order to deliver valid outcome recording. Likely, this may reflect the fact that, in contrast to a clinical trial, there is less inherent pressure to ‘produce’ a good outcome. Rather, if the outcome is insufficient for a patient’s needs, an alternative treatment will be contemplated.

We find that treatment outcomes are independent of the location performing the NB-UVB treatment. Although all four localities analysed are administrated by a single photobiology unit, individual staff differ. In addition, the treatment centers as such have a significantly varying overall throughput of patients, culminating in different levels of experience, given that the observational period of the study spans more than six years. In addition the unblinded assignment of treatment outcomes is open to inter-individually differing scores. Despite all these differences, both clinical outcomes as well as the number of treatment courses administered is very similar at different locations. These data strongly suggest that the observed efficacy rates are independent of local factors.

The statistically significant reduction in anti-histamine prescriptions is noteworthy. Pruritus is a common symptom in psoriasis and other treatments have been shown in randomized trials to positively impact on this important symptom. Even though pruritus as such is not routinely recorded in clinical practice, the reduction in the use of anti-histamines indicates that NB-UVB ameliorates this symptom. In a wider sense, these data therefore support the use of NB-UVB in non-psoriasis associated pruritus, which is often employed in clinical contexts not favorable to drug treatment.

In conclusion, we show that NB-UVB treatment leads to both a major sustained improvement as well as significant reduction in topical treatments in approximately 75% of patients treated for psoriasis. The data support efforts to increase access for this treatment.

## Supporting information

S1 Table The change in the number of patients receiving psoriasis-targeted topical treatment after one course of UVB phototherapy. (DOCX)

S2 Table Range of treatment number for UVB treatment episodes. (DOCX)

S3 Table The change in the number of patients receiving systemic psoriasis- treatment after one course of UVB phototherapy. (DOCX)

S1 Fig The change in prescriptions for psoriasis across all patients after, compared to before UVB treatment for psoriasis. (TIF)
