# Supplementary material for: Narrowband UVB treatment is highly effective and causes a strong reduction in the use of steroid and other creams in psoriasis patients in clinical practice
Source: PLoS One. 2017 Aug 3;12(8):e0181813. doi: 10.1371/journal.pone.0181813 (PMC5542593; doi:10.1371/journal.pone.0181813)
Supplement: S1 Table — (DOCX) [file pone.0181813.s001.docx]

Supporting Table S1. The change in the number of patients receiving psoriasis-targeted topical treatment after one course of UVB phototherapy.^1^

|  | Steroids | | Pso-Topicals | | Emollients | |
| --- | --- | --- | --- | --- | --- | --- |
| Outcome | before | after | before | after | before | after |
| 0/1 | 73.2 | 46.6 | 83.2 | 51.5 | 71.9 | 46.1 |
| 2 | 76.7 | 59.9 | 85.5 | 59.2 | 72.9 | 54.6 |
| 3/4/5 | 71.3 | 52.4 | 82.5 | 48.3 | 73.4 | 50.3 |

^1^ Outcome groups are “clear/minimal residual disease” (0/1), “moderate clearance” (2), “minimal improvement, no change, worsening” (3,4,5), respectively. Data shown represent the percentage of patients receiving prescriptions for n =1343 (outcome groups 0/1), n = 262 (outcome group 2), and n = 143 (outcome groups 3/4/5), respectively.
