# Supplementary material for: Narrowband UVB treatment is highly effective and causes a strong reduction in the use of steroid and other creams in psoriasis patients in clinical practice
Source: PLoS One. 2017 Aug 3;12(8):e0181813. doi: 10.1371/journal.pone.0181813 (PMC5542593; doi:10.1371/journal.pone.0181813)
Supplement: S2 Table — (DOCX) [file pone.0181813.s002.docx]

Supporting Table 2. Range of treatment number for UVB treatment episodes^1^

|  | Number of treatments | Number of weeks |
| --- | --- | --- |
| Average ± s.d. | 29.7 ± 10.5 | 9.9 ± 3.5 |
| Median | 28 | 9.3 |
| 10^th^ percentile | 18 | 6.0 |
| 90^th^ percentile | 42 | 14.0 |
| Maximum | 96 | 32 |
| Minimum | 10 | 3.3 |

^1^Data shown detail the number of actual narrowband UVB treatment sessions applied per treatment courses (n = 1749) administered as first-ever initial UVB treatment for psoriasis during the observational window specified in Methods. UVB treatment was administered at 3 sessions per week. The number of treatments exceeding 16 weeks was n = 74 (4.8 %).
