# Supplementary material for: Narrowband UVB treatment is highly effective and causes a strong reduction in the use of steroid and other creams in psoriasis patients in clinical practice
Source: PLoS One. 2017 Aug 3;12(8):e0181813. doi: 10.1371/journal.pone.0181813 (PMC5542593; doi:10.1371/journal.pone.0181813)
Supplement: S3 Table — (DOCX) [file pone.0181813.s003.docx]

Supporting Table S3. The change in the number of patients receiving systemic psoriasis- treatment after one course of UVB phototherapy.^1^

|  | Before UVB | | After UVB | |
| --- | --- | --- | --- | --- |
|  | N | % | N | % |
| Methotrexate | 50 | 2.9 | 61 | 3.5 |
| Acitretin | 4 | 0.2 | 8 | 0.5 |
| Cyclosporin | 1 | 0.06 | 4 | 0.23 |

^1^ N = 1749.
