# Supplementary figures and images for: Narrowband UVB treatment is highly effective and causes a strong reduction in the use of steroid and other creams in psoriasis patients in clinical practice
Source: PLoS One. 2017 Aug 3;12(8):e0181813. doi: 10.1371/journal.pone.0181813 (PMC5542593; doi:10.1371/journal.pone.0181813)

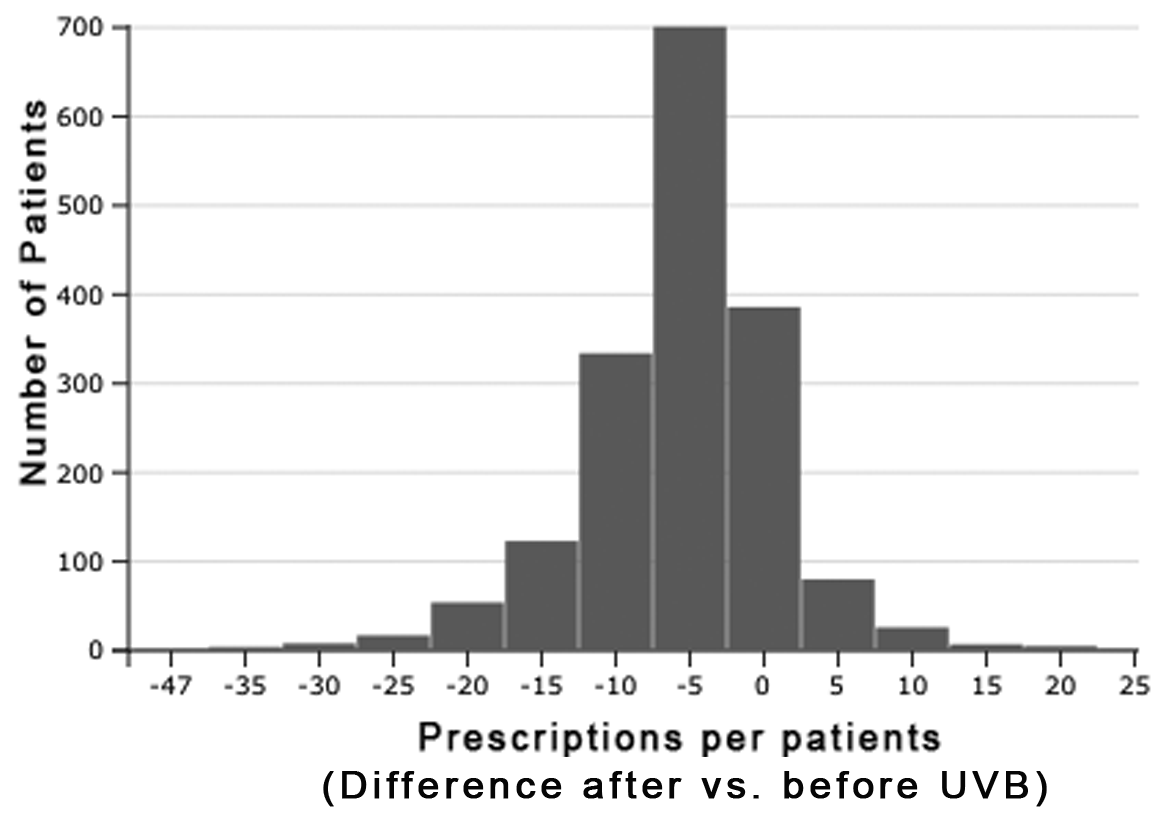

Supplement: S1 Fig — (TIF) [file pone.0181813.s004.tif]
